# Cardiovascular Disease Screening Programs in Low- and Middle-Income Countries: A Systematic Review

**DOI:** 10.1016/j.jacadv.2026.103048

**Published:** 2026-07-22

**Authors:** Eyad Jamileh, Ahmed T. Elmewafy, Mohammad Almeer, Priyanth Alaguraja, Sadeer Al-Kindi, Zainab Dakhil, Mirvat Alasnag, Jassim Al Suwaidi, Marwan M. Refaat, Mohamad B. Taha, Kaveh Hosseini, Wael Al Mahmeed, Amr Abdin, Mustafa Zakkar, Ibrahim Antoun

**Affiliations:** a Royal Blackburn Teaching Hospital; East Lancashire Hospitals Trust, Blackburn, England, United Kingdom; b School of Medicine, University of Sheffield, Sheffield, England, United Kingdom; c School of Medicine, University of Central Lancashire, Preston, England, United Kingdom; d Department of Cardiology, Kettering General Hospital, Kettering, United Kingdom; e Houston Methodist DeBakey Heart and Vascular Centre, Houston, Texas, USA; f Ibn Al-Bitar Cardiac Centre, University of Baghdad/Al-Kindy College of Medicine, Baghdad, Iraq; g Cardiac Centre, King Fahd Armed Forces Hospital, Jeddah, Saudi Arabia; h Hamad Medical Corporation, Doha, Qatar; i Department of Cardiology, American University of Beirut, Beirut, Lebanon; j Department of Cardiology, Copenhagen University Hospital - Herlev and Gentofte, Copenhagen, Denmark; k Center for Translational Cardiology and Pragmatic Randomized Trials, Department of Biomedical Sciences, Faculty of Health and Medical Sciences, University of Copenhagen, Denmark; l Cleveland Clinic Abu Dhabi, Abu Dhabi, United Arab Emirates; m Department of Internal Medicine III, Cardiology, Angiology, Intensive Care Medicine, Saarland University, Saarland University Medical Centre, Germany; n Department of Cardiac Surgery, University Hospitals of Leicester NHS Trust, Glenfield Hospital, Leicester, United Kingdom; o Department of Cardiovascular Sciences, Clinical Science Wing, University of Leicester, Glenfield Hospital, Leicester, United Kingdom

**Keywords:** cardiovascular disease, community health workers, diabetes, hypertension, low- and middle-income countries, screening

## Abstract

**Background:**

Cardiovascular disease (CVD) is the leading global cause of mortality and disproportionately affects low- and middle-income countries (LMICs). Although screening is essential for prevention and early detection, its effectiveness and implementation in LMICs remain unclear.

**Objectives:**

The objective of the study was to evaluate the diagnostic performance, detection yield, and linkage-to-care outcomes of CVD and major cardiometabolic risk-factor screening programs in LMICs.

**Methods:**

We conducted a systematic review in accordance with Preferred Reporting Items for Systematic Reviews and Meta-Analyses guidelines (PROSPERO, CRD420251241426). Databases were searched through November 2025. Observational and interventional studies assessing cardiovascular or major cardiometabolic screening programs in LMICs were included. Primary outcomes were detection rates and linkage to care. Secondary outcomes included risk-factor control, cardiovascular events, mortality, and cost-effectiveness. Owing to heterogeneity, results were synthesized narratively.

**Results:**

Seventeen studies from Asia, sub-Saharan Africa, and Latin America were included. Screening approaches comprised risk-prediction models, point-of-care testing, community health worker–led programs, and mobile health platforms. Diagnostic performance ranged from area under the curve 0.64 to 0.91. Locally adapted risk models generally outperformed imported scores, whereas simplified tools showed acceptable discrimination. Screening identified a substantial burden of undiagnosed disease, with up to 55% meeting hypertension criteria and approximately 40% classified as high risk for diabetes. However, considerable attrition occurred across the care cascade, with preventive service uptake ranging from 15% to 58% and confirmatory testing as low as 22%. Community health worker–led programs demonstrated >90% sensitivity and specificity compared with clinician assessment.

**Conclusions:**

CVD screening programs in LMICs can effectively identify unmet cardiometabolic risk, but weak linkage to care limits impact. Strengthening integrated screening-to-treatment pathways is essential to improve outcomes.

Cardiovascular disease (CVD) remains the leading cause of death worldwide, accounting for approximately 20.1 million deaths in 2021, with nearly 80% of these deaths occurring in low- and middle-income countries (LMICs).^1^^,^^2^ Early detection through screening is fundamental to prevention and risk reduction, particularly for modifiable conditions such as hypertension and diabetes.^3^

Despite this, access to and performance of screening programs vary widely across global settings. High-income countries (HICs) typically benefit from established primary care networks, structured referral systems, and greater health-system capacity for longitudinal follow-up. In contrast, LMICs frequently face structural constraints, including limited primary care access, reduced laboratory capacity, workforce shortages, and fragmented referral pathways.^4^, ^5^, ^6^

As a result, although the burden of CVD is greatest in LMICs, the effectiveness and performance of cardiovascular screening programs in these settings remain poorly understood. Beyond diagnostic accuracy, critical questions remain regarding detection yield, linkage to care, follow-up completion, and system-level feasibility. Evidence generated in high-income settings translate to LMIC contexts given substantial health care and socioeconomic differences.

To date, existing reviews on screening have often focused on specific conditions such as hypertension, with limited evidence addressing comprehensive cardiovascular screening or its effectiveness across diverse global settings. For example, systematic reviews of hypertension screening have primarily evaluated screening strategies and detection rates rather than broader population-level cardiovascular outcomes or downstream care processes.^7^ However, screening effectiveness in LMICs extends beyond diagnostic accuracy alone. Beyond identifying high-risk individuals, effective screening requires functional systems for awareness, referral completion, and sustained treatment engagement. In many LMIC settings, detection represents only the first step in a fragile care cascade, and improvements in diagnostic yield may not translate into meaningful reductions in morbidity or mortality. Understanding where breakdowns occur along this continuum, and which screening models successfully convert case identification into sustained care, is therefore essential. Accordingly, a systematic review is needed to evaluate not only the diagnostic performance of cardiovascular screening programs in LMICs, but also their implementation characteristics, program uptake, linkage to care, and clinical impact. Such evidence is critical to inform context-appropriate screening strategies and guide policy development aimed at reducing the global burden of CVD.

This systematic review, therefore, aims to evaluate the effectiveness and implementation of CVD screening programs in LMICs. The population includes adults and community-based populations in LMICs undergoing screening for CVD or major cardiometabolic risk factors. Interventions included community, primary care or facility-based screening programs employing clinical measurements, risk prediction tools, point-of-care diagnostics, community health worker (CHW)-led models, or mobile health (mHealth) technologies. Comparators included usual care, alternative screening strategies, and, where applicable, no formal comparator. Primary outcomes were detection yield (identification of previously undiagnosed CVD or cardiometabolic risk factors) and linkage to care, including referral completion, confirmatory testing, and treatment initiation. Secondary outcomes included program uptake, risk-factor control, cardiovascular events, mortality, and cost-effectiveness.

## Methods

This systematic review followed the Preferred Reporting Items for Systematic Reviews and Meta-Analyses (PRISMA) guidelines.^8^ A study protocol conforming to the PRISMA guidelines was registered with the International Prospective Register of Systematic Reviews (PROSPERO ID: CRD420251241426),^9^ and the PRISMA checklist is reported in our Supplemental Appendix.

### Eligibility criteria

This study aimed to evaluate the effectiveness and performance of CVD screening programs implemented in LMICs as classified by World Bank income categories at the time of study conduct. We synthesized evidence from individually randomized controlled trials, cluster-randomized trials, quasi-experimental studies, prospective cohort studies, retrospective cohort studies, cross-sectional studies, and program evaluations that evaluated screening interventions for CVD or major cardiovascular risk factors, including hypertension and diabetes. Full-text articles and conference abstracts were eligible where sufficient outcome data were available. The registered protocol specified inclusion of adults aged ≥18 years. During study selection, we did not apply a strict age restriction because some community-based LMIC screening programs reported mixed or broad population groups. No restrictions were placed on sex or baseline cardiovascular risk.

Studies were eligible for inclusion if they evaluated a defined cardiovascular screening program and reported quantifiable screening-related outcomes. Screening interventions could include community-based, population-level, or primary care-based programs using clinical measurements, risk assessment tools, or combined approaches to identify CVD or major cardiovascular risk factors.

To ensure generalizability, no restrictions were placed on participant demographics, including age, sex, or baseline cardiovascular risk. Studies of both asymptomatic screening populations and mixed populations were considered. Only articles and abstracts published in English were included.

Studies were excluded if they were case reports, single-arm observational studies without a defined screening intervention, narrative reviews, or conference abstracts lacking sufficient outcome data. In addition, studies conducted exclusively in HICs or studies that did not report the prespecified outcomes were excluded.

### Primary outcomes

The primary outcomes of this review were program effectiveness outcomes, including detection rates of previously undiagnosed CVD or major cardiovascular risk factors identified through screening programs and linkage-to-care rates following screening; and implementation outcomes, including program uptake, number of individuals screened, screening completion, and referral completion. Linkage-to-care outcomes included attendance at follow-up clinical visits, completion of confirmatory testing or referrals, and initiation of appropriate treatment where reported.

### Secondary outcomes

Secondary outcomes included changes in cardiovascular risk-factor control following screening, such as blood pressure (BP), blood glucose, or lipid levels; cardiovascular events including myocardial infarction and stroke; all-cause or cardiovascular mortality; cost-effectiveness; workforce implementation characteristics (including CHW or provider-led delivery); feasibility, acceptability, and health-system barriers were reported.

### Literature search strategy

A comprehensive literature search was conducted by two independent reviewers across multiple electronic databases, including MEDLINE (via PubMed), EMBASE, CINAHL, Web of Science, and the Cochrane Central Register of Controlled Trials (CENTRAL). Search results retrieved from each database were independently reviewed by two reviewers to ensure completeness and consistency. Any discrepancies in retrieved records or eligibility for inclusion at this stage were resolved through discussion, with consultation from a third reviewer where necessary. The final search was completed on November 22, 2025, after the initiation of the review. The complete search strategies are fully described in the Supplemental Appendix. In addition to database searches, gray literature and ongoing or unpublished studies were identified through searches of the World Health Organization International Clinical Trials Registry Platform, ClinicalTrials.gov, and the International Randomized Controlled Trial Number Register. To ensure thorough coverage, reference lists of all included studies and relevant systematic reviews were manually screened for additional eligible articles.

### Study selection

Two independent reviewers, A.E. and P.A. (blinded to each other’s assessments), screened all retrieved studies for eligibility based on titles and abstracts using predefined inclusion and exclusion criteria. Full-text articles for potentially eligible studies were subsequently retrieved and independently assessed in duplicate by the same reviewers using a standardized screening form based on the prespecified eligibility criteria. If discrepancies in the selection process were identified, they were resolved through discussion or consultation with a third reviewer, E.J.

### Data extraction and management

A standardized data extraction spreadsheet was developed using Microsoft Excel, informed by the Cochrane data collection form for systematic reviews of public health and screening interventions. The extraction sheet was pilot-tested to ensure the consistency, clarity, and reproducibility of data entry. Two independent reviewers extracted data from all eligible studies, with disagreements resolved through discussion and consensus.

Extracted variables included key study characteristics, patient demographics, details of the screening intervention, and all prespecified primary and secondary outcomes. Where reported, information on screening setting, personnel involved, referral pathways, and follow-up mechanisms were also collected.

### Data synthesis

Due to heterogeneity, data were synthesized narratively. Across the included studies, methodological and contextual heterogeneity precluded a pooled meta-analysis. Screening strategies, health care settings, population characteristics, and outcome reporting varied substantially between studies. Given these differences in study design, outcome definitions, and reporting formats, statistical pooling was not appropriate. Therefore, results were synthesized narratively. In addition to modality-based categorization, findings were interpreted across the screening continuum, including: 1) detection performance; 2) awareness and initial classification; 3) referral and confirmatory testing; and 4) treatment initiation or follow-up engagement, to identify structural determinants of program effectiveness.

### Risk of bias and quality assessment

The methodological quality of randomized studies was assessed using the Cochrane Risk of Bias 2 (RoB 2) tool, whereas nonrandomized studies were assessed using the Risk Of Bias In Nonrandomized Studies of Interventions (ROBINS-I) tool. Risk-of-bias findings were summarized separately according to study design. Two reviewers assessed methodological quality independently, with disagreements resolved through discussion and consensus, or consultation with a third reviewer where necessary.

## Results

A total of 879 records were identified through database searching, supplemented by gray literature searches, which yielded no additional eligible studies. After duplicate removal, 758 unique records underwent title and abstract screening. Of these, 738 records were excluded, including reviews or case reports (n = 324), unrelated topics (n = 79), abstract-only publications (n = 87), and ineligible populations/settings (n = 248), primarily studies conducted in HICs, studies focused on noncardiovascular screening programs, or populations outside the review scope such as exclusively hospital-based diagnostic cohorts without screening relevance.

**Figure 1:**
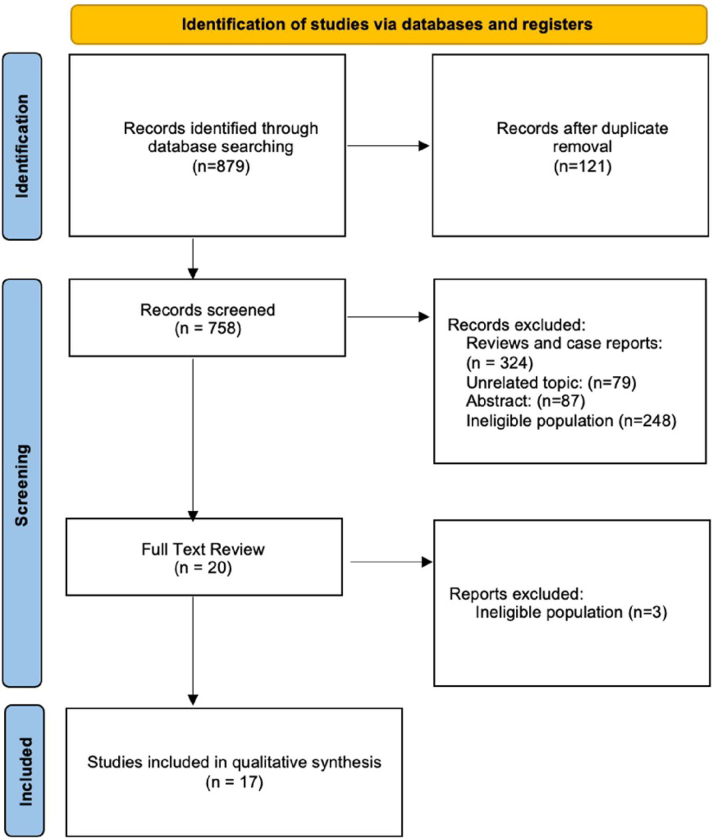
**Study Selection Flow Diagram** Preferred Reporting Items for Systematic Reviews and Meta-Analyses flow diagram showing the identification, screening, eligibility assessment, and final inclusion of studies in the systematic review.

Twenty full-text articles were assessed for eligibility, with 3 further studies excluded due to ineligible settings or interventions not aligned with cardiovascular screening in LMIC contexts. Reasons for full text exclusion are detailed in the Supplemental Appendix. Ultimately, 17 studies were included in the qualitative synthesis. This is summarized in Figure 1.

These 17 studies evaluated cardiovascular screening strategies across LMICs, including settings in Asia (Cambodia, rural China, India, Nepal, and Pakistan), sub-Saharan Africa (Kenya, Tanzania, Rwanda, and Eswatini), Latin America (Brazil, Guatemala, and Mexico), and multinational cohorts spanning up to 56 LMICs. Sample sizes ranged from 268 participants to 1.2 million individuals. Screening modalities included risk-prediction models, point-of-care diagnostic testing, CHW-led programs, and mHealth platforms. Across settings, screening strategies varied substantially in diagnostic performance, detection yield, and progression through follow-up care. The characteristics of each study are summarized in Table 1.

**Table 1:** Characteristics of Included Studies

First Author, Year	Country/Setting	Design	Population	Cadre	Setting	Intervention/Screening Activity	Comparator	Condition/Risk Category	Key Outcomes
Rahim et al, 2023^16^	44 LMICs	Pooled cross-sectional survey analysis	Adults >25 y, nonpregnant, without diabetes; n = 145,739	Health systems/health care professionals	Nationally representative community surveys	Assessment of diabetes risk and receipt of prevention activities	None	High diabetes risk: IFG/prediabetes or overweight/obesity	40.6% high risk; <50% received prevention activities
Sudharsanan et al, 2020^28^	South Africa	Regression discontinuity cohort analysis	Adults ≥30 y in national panel survey	Fieldworkers	Home/community	Home-based BP measurement and advice to seek care if BP high	Just below BP threshold	Hypertension screening; BP ≥140/90	Reduced SBP in women and younger men; no DBP effect
Edward et al, 2020^24^	Tanzania	Exploratory observational + prepost training study	Adult outpatient/camp attendees; 69 observations; 33 providers	Doctors, nurses, other providers	Clinic and screening camps	BP screening quality and counseling; training videos	Preprovider vs postprovider knowledge	Hypertension screening/management	Poor counseling; improved knowledge after videos
Etyang et al, 2019^14^	Kenya	Diagnostic accuracy study	Random adult population sample; n = 982	Study staff	Study clinics/community recruitment	Unattended automated office BP	24-h ABPM reference standard	Hypertension diagnosis	uAOBP had modest accuracy; sensitivity 44%–67% depending cutoff
Zhao et al, 2013^12^	Rural China	Cross-sectional diagnostic accuracy study	Adults >20 y; n = 993	Study/research staff	Rural community	Fasting capillary glucose screening	FPG and OGTT	Diabetes/prediabetes	FCG AUC 0.88 for diabetes; acceptable rural screening tool
Makusidi et al, 2013^29^	Nigeria	Descriptive cross-sectional screening program	World Kidney Day participants; n = 535	Health screening team	Community screening event	BP, glucose, BMI, WHR, urinalysis	None	HTN, diabetes, obesity, proteinuria	HTN 30.2%; diabetes 6%; proteinuria 17.9%; low awareness
Ishaq et al, 2019^17^	Pakistan	Cross-sectional screening campaign	Adults ≥18 y; n = 5,333	Trained volunteers	Public/community screening camps	May Measurement Month BP screening	None	Hypertension ≥140/90 or treatment	Hypertension prevalence 55.2%
Gaziano et al, 2015^22^	Bangladesh, Guatemala, Mexico, South Africa	Observational validation study	Adults 35–74 y without known HTN/diabetes/CVD; n = 4,049	CHWs	Community/home-based	Noninvasive CVD risk score	Physician/nurse risk score	5-y CVD risk; >20% high risk	CHW–professional agreement 96.8%; κ = 0.948
Mannik et al, 2018^20^	Rural Kenya	mHealth feasibility/implementation study	Adults >40 years; n = 2,865	CHWs	Rural community	AFYACHAT SMS CVD risk tool	None	10-y CVD risk: green <10%, yellow 10%–<20%, orange 20%–<30%, red ≥30%	Feasible; 23% hypertension; 97% low risk
Niyibizi et al, 2023^19^	Rwanda	Action research/implementation study	Adults 35–74 y; n = 995	CHWs, nurses	Rural and urban communities	KoboCollect BMI-based CVD risk screening and referral	Nurse-generated score	10-y CVD risk: low <10%, moderate 10%–<20%, high ≥20%	CHW–nurse scores correlated; referral compliance 68.8%
Boutilier et al, 2021^10^	Hyderabad, India	Machine-learning model development and validation	Community-screened individuals; n = 2,278	CHWs	Door-to-door and camp-based screening in urban slums	Machine-learning risk stratification using demographic, questionnaire, anthropometric, glucose, and BP data	Established and retrained US- and UK-derived risk scores	Diabetes and hypertension risk	Random-forest models improved AUC for diabetes (0.910 vs 0.671) and hypertension (0.792 vs 0.698) and reduced false-negative classifications.
Kirschbaum et al, 2021^11^	56 LMICs	Pooled cross-sectional survey analysis	Adults from nationally representative surveys; n = 1,170,629	Trained national survey fieldworkers	Nationally representative household surveys	Hypertension-targeting models using age, BMI, sex, and smoking status	Age alone versus models adding BMI, sex, and smoking	Hypertension	Model AUC ranged from approximately 0.64 to 0.85; adding BMI, sex, and smoking to age improved AUC by only approximately 0.05.
Rawal et al, 2022^23^	Nepal	Community screening validation study	Adults screened for CVD risk	Female community health volunteers	Community	CVD risk screening by FCHVs	Doctor-generated scores	10-y CVD risk	Good/substantial agreement with doctors
Storey et al, 2018^13^	Phnom Penh, Cambodia	Prospective cross-sectional diagnostic-accuracy study	Community-dwelling adults ≥18 years; n = 1,289	Participants and trained study staff	Community	Self-administered urine glucose strips; HbA1c and capillary fasting glucose also assessed	Composite reference standard using fasting glucose and 2-hour OGTT glucose	Diabetes	Diabetes was identified in 18%; urine strips had 14% sensitivity and 99% specificity, missing 201 cases.
Forsvall et al, 2006^15^	Bahia, Brazil	Diagnostic device evaluation and validation study	Patients undergoing BP assessment; n = 268	Two trained operators	Outpatient clinics	Rastreometro simplified BP-screening device	Standard auscultatory BP measurement	Hypertension	Sensitivity 95.1%, specificity 63.1%, PPV 62.4%, and NPV 95.3%; specificity was lower among treated patients.
Palma et al, 2018^18^	Manzini, Swaziland/Eswatini	Observational time-motion and implementation study	HIV-clinic patients aged ≥40 years; 1,826 screened; 172 visits observed	Trained HIV-clinic staff	Urban HIV clinic	CVD risk-factor screening integrated into routine HIV visits, including BP, glucose, cholesterol, smoking, and risk assessment	HIV-clinic visits without integrated CV screening	Hypertension, diabetes, dyslipidaemia, smoking, and overall CV risk	39% had ≥1 CV risk factor; screening increased median visit duration from approximately 4 to 15 minutes but was highly acceptable.
Pastakia et al, 2013^21^	Webuye, western Kenya	Comparative feasibility study	Adults >18 years; n = 582: 236 home-based and 346 community-based	HIV counsellors; district-hospital nurses and clinical staff	Home and community screening	BP and random-glucose screening with referral for confirmatory testing	Home-based versus community-based screening	Hypertension and diabetes	Home screening detected more elevated glucose (23% vs 8%); community screening detected more elevated SBP (10% vs 6%). Confirmatory follow-up was low at 22%–31%.

ABPM = ambulatory blood pressure monitoring; AFYACHAT = a short message service–based mobile health cardiovascular risk-screening tool; ART = antiretroviral therapy; AUC = area under the curve; BMI = body mass index; BP = blood pressure; CHW = community health worker; CV = cardiovascular; CVD = cardiovascular disease; DBP = diastolic blood pressure; FCG = fasting capillary glucose; FCHV = female community health volunteer; FPG = fasting plasma glucose; HbA1c = glycated hemoglobin; HIV = human immunodeficiency virus; HTN = hypertension; IFG = impaired fasting glucose; LMICs = low- and middle-income countries; mHealth = mobile health; NPV = negative predictive value; OGTT = oral glucose tolerance test; PPV = positive predictive value; SBP = systolic blood pressure; SMS = short message service; uAOBP = unattended automated office blood pressure; WHR = waist-to-hip ratio.

### Risk-prediction approaches

The performance of the prediction model demonstrated marked variability across LMIC settings, with locally adapted approaches outperforming imported or noncontextualized risk scores. In Hyderabad, India, Boutilier et al demonstrated that traditional diabetes risk scores (American Diabetes Association and UK-based) performed modestly (area under the curve [AUC]: 0.64-0.67). In contrast, a locally trained random-forest model achieved an AUC of 0.910, representing a 35.5% improvement over the best retrained baseline (AUC: 0.671).^10^ When restricted to questionnaire-only variables, performance retained approximately 87% of the full-feature AUC.^10^ At a larger scale, Kirschbaum et al^11^, analyzing 1.2 million adults across 56 LMICs, reported that age alone demonstrated strong discriminatory capacity for hypertension, with inclusion of body mass index (BMI), sex, and smoking status improving AUC by only 0.05.

Together, these studies indicate that discriminatory performance ranged from modest (AUC: ∼0.64) to high (AUC: ∼0.91),^10^^,^^11^ with locally adapted or simplified approaches achieving acceptable predictive accuracy. However, few studies evaluated whether improved discrimination translated into improved referral completion or treatment uptake. Thus, although locally adapted models enhanced detection accuracy, their downstream implementation impact remains incompletely characterized.

### Point-of-care diagnostic tools

Point-of-care tools demonstrated marked heterogeneity in accuracy. In rural China (n = 993), Zhao et al^12^ reported that capillary glucose testing achieved an AUC of 0.88, with 84.2% sensitivity and 79.3% specificity at a threshold of 5.65 mmol/L. Fasting plasma glucose demonstrated an AUC of 0.92, with 82.5% sensitivity and 98.3% specificity at 6.51 mmol/L, indicating comparable overall discriminatory performance.^12^ In contrast, Storey et al^13^ evaluated self-administered urine glucose strips in Cambodia and observed low sensitivity (14%) despite high specificity (99%), corresponding to an estimated false-negative rate of approximately 86%.

Hypertension screening tools showed similar variability. In Kenya, Etyang et al^14^ found that unattended automated office BP yielded mean systolic readings comparable to ambulatory monitoring (difference: 0.6 mmHg), yet with wide limits of agreement (−39 to +40 mmHg), resulting in substantial misclassification. Conversely, in rural Brazil (n = 268), Forsvall et al reported that the Rastreometro (a low-cost, sticker-modified aneroid sphygmomanometer) achieved sensitivity of 95.1% and specificity of 63.1% (positive predictive value 62.4%; negative predictive value 95.3%), although specificity declined to 33% among individuals receiving antihypertensive therapy compared with 78% in untreated participants.^15^

Overall, screening tool performance ranged from high sensitivity (>95%) to markedly limited sensitivity (14%), reflecting variability across devices and contexts.^13^, ^14^, ^15^ Notably, devices with lower sensitivity risk substantial underdetection, whereas tools with limited specificity may increase inappropriate referrals, placing strain on already resource-constrained health systems. These findings suggest that device selection has implications not only for diagnostic accuracy but also for downstream system burden.

### Detection yield and burden identified

Across studies, screening programs frequently identified substantial proportions of high-risk or previously undiagnosed individuals. In a multinational cohort of 145,739 adults across 44 LMICs, Rahim et al^16^ reported that 40.7% (95% CI: 38.5%-42.8%) were classified as high risk for type 2 diabetes based on impaired fasting glucose, glycosylated hemoglobin–defined prediabetes, or obesity. Similarly, digital BP screening of 5,333 adults in Pakistan by Ishaq et al^17^ identified 55% as hypertensive. In Eswatini, integration of cardiovascular screening into HIV clinics by Palma et al^18^ identified cardiovascular risk factors in 39% of 1,826 participants.

Risk stratification approaches yielded varying proportions of high-risk classification. In Rwanda, Niyibizi et al^19^ reported that 23.9% of participants were categorized as having ≥10% 10-year cardiovascular risk, whereas in rural Kenya, Mannik et al^20^ identified 3% of screened individuals as having ≥10% 10-year risk using a mHealth platform. Home-based screening strategies were associated with increased case identification; Pastakia et al^21^ found that home-based diabetes screening identified 3.5 times more individuals with elevated glucose than community-based approaches. Although detection yields were consistently high, multiple studies reported limited confirmatory testing, incomplete attendance at referral, and variable treatment initiation. This discrepancy suggests that identifying previously undiagnosed individuals does not automatically translate into effective management, particularly where confirmatory testing and longitudinal follow-up are limited.

### Follow-up and care cascade

Despite high rates of detection, follow-up engagement and preventive service uptake were limited. In the multinational analysis (44 LMICs) by Rahim et al, reported uptake of prevention services ranged from 37% to 42% overall: 40% received physical-activity counseling, 37% received weight-loss counseling, 42% received dietary counseling, and 37% received blood-glucose screening.^16^ Coverage was substantially lower in low-income countries (15% to 20%) compared with upper-middle-income countries (50% to 58%).^16^ Following the identification of elevated glucose in community screening, Pastakia et al^21^ reported that only 22% of individuals returned for confirmatory testing despite phone-based reminders. In Rwanda, Niyibizi et al^19^ observed that 68.8% of individuals referred after CHW screening attended health facilities; 30.8% were diagnosed with hypertension and/or diabetes and initiated on medication. Postreferral, 54.7% reported follow-up, and 90.5% expressed satisfaction with care received, with financial and time constraints identified as key barriers to referral uptake.^19^ Across various settings, attrition from initial screening to confirmed diagnosis frequently exceeded 40%, and in some community-based programs it surpassed 75%. These findings indicate that referral infrastructure and follow-up mechanisms (rather than screening accuracy alone) represent the dominant constraints on program effectiveness in LMIC contexts.

**Figure 2:**
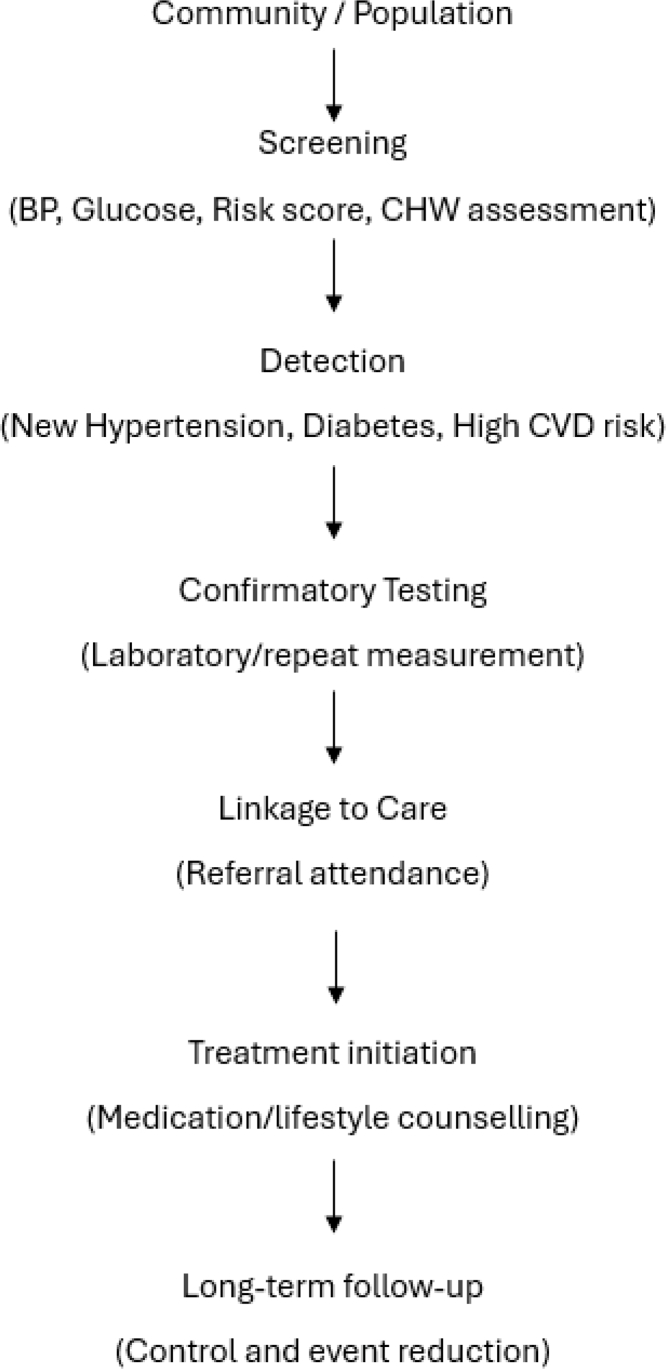
**Screening-to-Care Cascade for Cardiovascular Disease Programs in Low- and Middle-Income Countries** BP = blood pressure; CHW = community health worker; CVD = cardiovascular disease.

To facilitate interpretation of the attrition observed across studies, the screening-to-care pathway is conceptualized as a sequential cascade from screening and detection through linkage, treatment initiation, and long-term follow-up (Figure 2). Across LMIC settings, the largest losses occurred at the linkage and confirmatory testing stages, particularly in low-income contexts.

### Workforce and implementation models

Across Bangladesh, Guatemala, South Africa, and Mexico, Gaziano et al^22^ reported 96.8% agreement (95% CI: 0.936-0.961) between CHW and health care professional cardiovascular risk assessments. Similarly, in Nepal, Rawal et al^23^ demonstrated 90% sensitivity and 97% specificity, with 94.5% overall agreement (κ = 0.77; 95% CI: 0.705-0.835) between CHWs and health care professionals. Importantly, CHW-based models demonstrated high diagnostic concordance with clinician assessments and were associated with measurable referral attendance in several settings, indicating that workforce design may influence progression through the care cascade.

### Infrastructure and provider-level constraints

Implementation barriers were identified across patient, provider, and health-system levels. Rahim et al^16^ described multilevel constraints across 44 LMICs, including low patient awareness, limited clinician familiarity with guideline-based prevention strategies, and insufficient laboratory capacity to support confirmatory testing and risk stratification. At the provider level, Edward et al^24^ similarly identified deficiencies in BP measurement technique and counseling practices during 69 observed consultations in Tanzania; brief instructional videos improved knowledge in selected domains but did not fully address technical gaps. In rural Kenya, Mannik et al^20^ reported that mHealth-facilitated risk stratification of 2,865 community members was feasible but constrained by power interruptions and SMS transmission delays.

In contrast, integration of cardiovascular screening into existing service platforms demonstrated high patient acceptability. In Eswatini, Palma et al^18^ reported that 98% of participants experienced no unpleasantness and 63% were willing to allocate ≥10 minutes annually for screening, suggesting that embedding screening within routine care may mitigate some engagement barriers. Collectively, these findings indicate that structural and provider-level constraints, including laboratory capacity, measurement—technique variability— and digital infrastructure reliability, exert a greater influence on screening effectiveness than on risk-model discrimination alone.

### Methodological quality and risk of bias assessment

**Figure 3:**
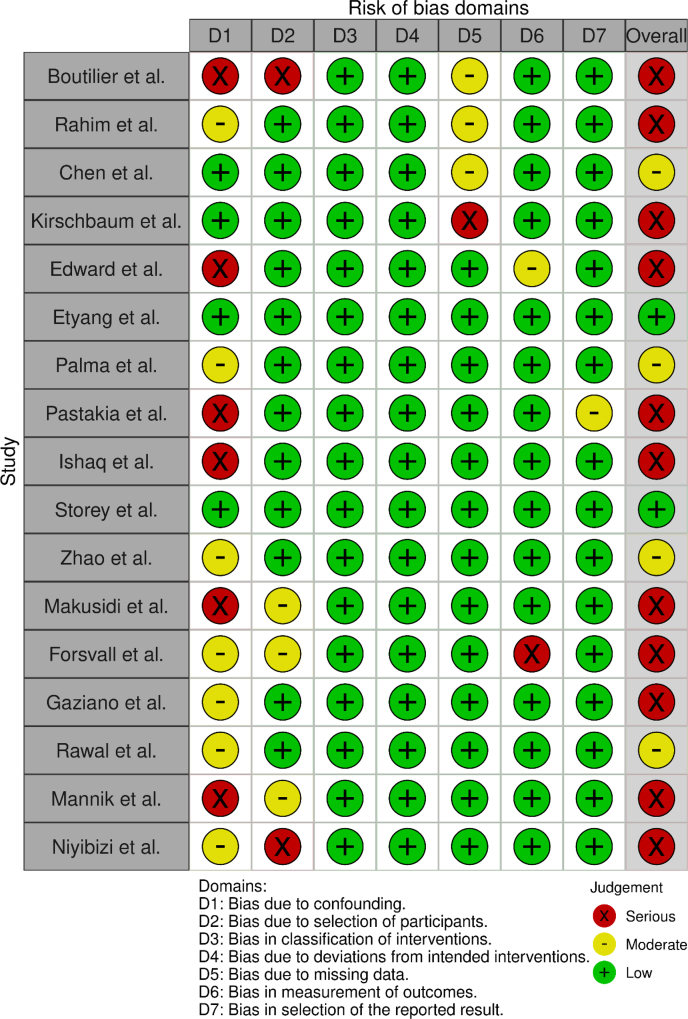
**Risk of Bias Across Included Studies** Traffic light plot summarizing the Risk Of Bias In Nonrandomized Studies of Interventions assessment. D1, bias due to confounding; D2, bias in selection of participants; D3, bias in classification of interventions; D4, bias due to deviations from intended interventions; D5, bias due to missing data; D6, bias in measurement of outcomes; and D7, bias in selection of the reported result. Green indicates low risk, yellow indicates moderate risk, and red indicates serious risk of bias.

The methodological quality of the included studies was variable. Overall, several studies were judged to have a serious risk of bias, most commonly arising from confounding (D1) and participant selection (D2), reflecting the predominantly observational nature of the evidence and the use of nonrandomized screening cohorts. Missing data (D5) also contributed to moderate or serious risk in a subset of studies. In contrast, most studies were assessed as low risk in domains relating to classification of interventions (D3), deviations from intended interventions (D4), measurement of outcomes (D6), and selection of reported results (D7), suggesting generally consistent outcome assessment and reporting practices. Overall risk of bias was frequently driven by structural limitations inherent to real-world implementation studies in LMIC settings rather than by flaws in outcome measurement. This is summarized in Figure 3.

## Discussion

This systematic review synthesizes evidence from 17 studies evaluating CVD screening strategies across diverse LMIC settings. Across heterogeneous contexts, consistent themes emerged: substantial variability in diagnostic performance; identification of large reservoirs of previously undiagnosed or high-risk individuals; strong agreement between CHW-led and clinician assessments; and pronounced attrition across the care cascade following detection (Central Illustration). Collectively, these findings suggest that the primary implementation barrier in LMIC screening systems lies not only in risk identification, but in ensuring awareness, referral completion, and sustained linkage to care. Across diverse settings, the dominant implementation failure was not an inability to detect high-risk individuals, but a failure to convert detection into sustained care.

**Central Illustration:**
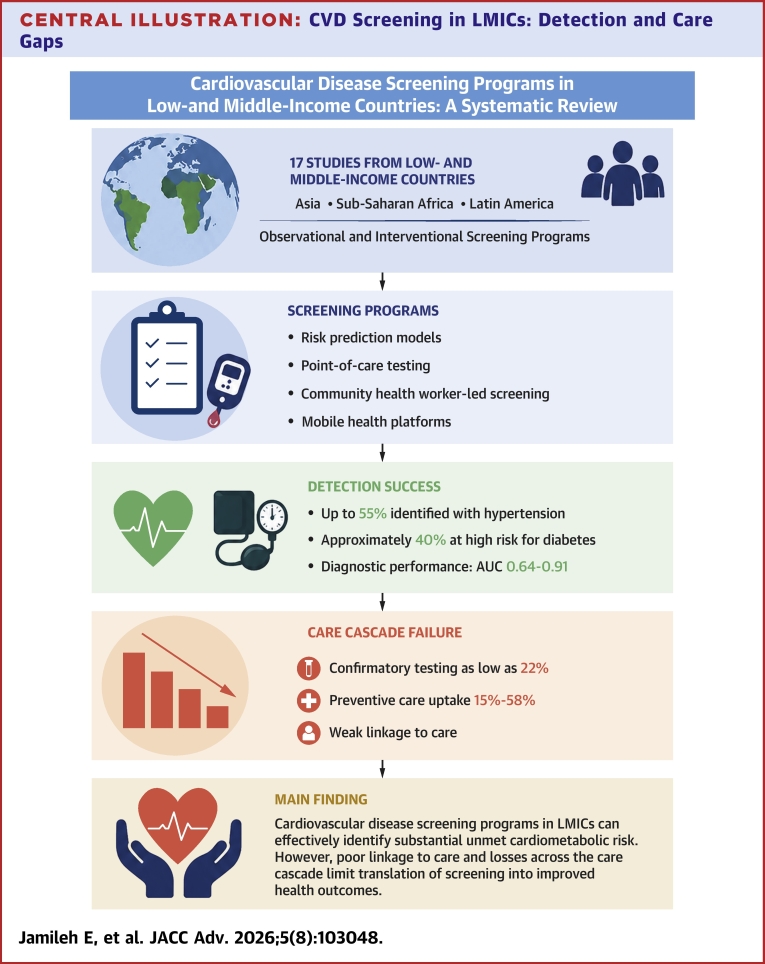
**CVD Screening in LMICs: Detection and Care Gaps** Diverse screening modalities, including risk tools, point-of-care diagnostics, CHW-led programs, and mHealth platforms, demonstrate substantial capacity to identify hidden cardiometabolic burden in LMICs, with high hypertension and diabetes risk detection, acceptable diagnostic performance, and strong CHW-clinician agreement. However, major attrition occurs across the screening-to-care cascade, particularly during confirmatory testing, referral, treatment initiation, and long-term follow-up. Financial constraints, weak infrastructure, limited laboratory capacity, geographic barriers, and workforce shortages are major implementation barriers. These findings indicate that screening effectiveness depends not only on detection, but on linkage-to-care and downstream system capacity. Sustainable impact requires locally validated tools, CHW workforce expansion, integrated primary care pathways, strengthened referral systems, digital follow-up, and broader health system strengthening to translate detection into improved cardiovascular outcomes. AUC = area under the curve; LMICs = low- and middle-income countries.

Substantial heterogeneity was observed across regions and income strata. Studies conducted in low-income settings consistently demonstrated lower linkage-to-care and preventive service uptake than in upper-middle-income countries. However, differences likely reflect not only economic status but also geographic, health-system, and sociocultural variation, which remain incompletely characterized in current screening literature.

Given that over 80% of premature cardiovascular mortality now occurs in LMICs, these findings have implications far beyond local screening design and directly inform global strategies to reduce noncommunicable disease inequities under the Sustainable Development Goals and World Health Organization Global Action Plan for NCD prevention.^1^^,^^2^ In this context, cardiovascular screening in LMICs should be viewed not solely as a diagnostic exercise, but as a core component of global health system strengthening aimed at reducing preventable morbidity, mortality, and economic productivity loss.

To contextualize these findings, cardiovascular screening in LMICs can be conceptualized as a multistage care cascade comprising: 1) population engagement and screening uptake; 2) risk detection; 3) confirmatory testing; 4) referral attendance; 5) treatment initiation; and 6) long-term risk factor control. Across the included studies, the most substantial losses consistently occurred beyond the detection stage, particularly during referral completion and treatment initiation. This cascade perspective clarifies that screening effectiveness is determined not only by diagnostic accuracy, but by the integrity of downstream care systems.

From a policy perspective, this cascade framework suggests that national screening programs should be evaluated not only by the number of individuals screened, but by cascade completion metrics including referral attendance, treatment initiation, and long-term control. Policy frameworks that prioritize screening coverage alone risk overestimating program success while neglecting the downstream system investments required to achieve population-level cardiovascular benefit.^5^

A central finding of this review is that screening performance is highly context dependent. The discriminatory capacity of risk prediction tools varied considerably across settings, with reported AUC values ranging from modest to very good. Models developed or recalibrated using local population data consistently outperformed risk scores derived in high-income settings. For example, in Hyderabad, a locally trained random forest model markedly outperformed established Western-derived diabetes risk scores, underscoring the importance of tailoring prediction tools to the epidemiological and demographic characteristics of the target population.^10^ Similarly, in multinational analyses, age alone demonstrated strong discriminatory capacity for hypertension, whereas the addition of BMI, sex, and smoking status provided only marginal incremental benefit.^11^ These findings suggest that effective risk stratification in LMICs does not necessarily require complex, data-intensive models. Locally calibrated tools based on simple, readily available variables may offer a better balance among discrimination, feasibility, and scalability. Overly complex or laboratory-intensive algorithms may hinder uptake at the community level and limit scalability, thereby constraining awareness and early referral.

A similar variability was observed in point-of-care diagnostic tools. In rural China, capillary glucose and fasting plasma glucose testing demonstrated strong discriminatory performance, supporting their use in settings with limited laboratory infrastructure.^12^ In contrast, self-administered urine glucose strips exhibited extremely low sensitivity, missing the majority of true cases despite high specificity.^13^ Hypertension screening devices likewise demonstrated heterogeneity in diagnostic performance.^14^^,^^15^ Such variability has direct implications for detection: underestimation may delay referral and treatment initiation, whereas overestimation may generate unnecessary strain on limited health system resources. These findings underscore that affordability alone is insufficient; screening devices must undergo rigorous local validation to ensure reliable case identification and appropriate referral.

Importantly, screening initiatives frequently identified a substantial proportion of individuals who were previously undiagnosed or at high cardiovascular risk. In multinational cohorts, nearly 40% of adults were classified as high risk for diabetes,^16^ and in several national and regional programs, more than half met diagnostic thresholds for hypertension.^17^^,^^18^ These figures highlight a considerable and often under-recognized burden of cardiometabolic disease across LMICs. However, identification alone does not translate into improved outcomes. Without structured referral pathways, accessible pharmacotherapy, and sustained follow-up, screening risks functioning as a diagnostic endpoint rather than an effective prevention strategy.

Screening devices must undergo local validation within the populations in which they are deployed to minimize systematic underdiagnosis or overdiagnosis and to ensure that detection strategies strengthen, rather than destabilize, downstream care pathways.

Attrition along the care cascade emerged as a consistent and critical limitation. Although programs frequently succeeded in identifying high-risk individuals, progression to confirmatory assessment and preventive care was markedly lower. Uptake of preventive services ranged from 37% to 42%, with coverage as low as 15% to 20% in low-income settings.^16^ In some community programs, only 22% of participants attended confirmatory follow-up after abnormal results, despite reminders.^21^ Even where referral attendance appeared relatively strong, such as CHW-led referral in Rwanda (68.8% attendance), only one-third were ultimately diagnosed and initiated on treatment.^19^

These findings suggest that the effectiveness of screening models in LMICs is primarily constrained by weaknesses in referral systems, patient awareness, and continuity of care rather than by detection alone. Financial barriers, indirect costs, limited health literacy, inadequate laboratory infrastructure, medication supply constraints, and provider-level gaps in preventive counseling all contributed to drop-off.^20^^,^^24^ Strengthening referral mechanisms, reducing structural barriers to follow-up, and embedding screening within continuous care platforms are therefore essential to improve real-world impact.

Encouragingly, CHW-led screening models demonstrated strong agreement with clinician assessments across multiple settings.^19^^,^^22^^,^^23^ High sensitivity, specificity, and inter-rater agreement indicate that task-shifting can effectively expand detection capacity in workforce-limited environments. Screening programs integrated into existing primary care or chronic disease platforms also demonstrated greater acceptability and operational feasibility than standalone campaigns. These models appear to improve awareness, facilitate completion of referrals, and enhance sustainability when supported by simplified risk tools and structured supervision.

At the health-system level, implementation of effective screening will also require alignment with universal health coverage policies, essential medicines programs, and primary care financing reforms. Screening programs that identify high-risk individuals without ensuring affordable access to antihypertensives, diabetes medications, and longitudinal follow-up may widen awareness without improving outcomes, thereby limiting public health return on investment.

Screening programs integrated into existing primary care or chronic disease platforms also demonstrated greater acceptability and operational feasibility than standalone campaigns. Models embedded within existing chronic care systems demonstrated greater feasibility and acceptability than standalone screening campaigns.

Nevertheless, implementation barriers remain substantial. Equipment unreliability, inconsistent measurement techniques, limited laboratory capacity, power interruptions, and supply chain instability frequently undermined screening effectiveness. These findings reinforce that screening performance reflects broader health system readiness as much as algorithm design. Without investment in infrastructure, training, and supply stability, even well-designed screening models may fail to translate into meaningful improvements in cardiovascular outcomes.

Across included studies, screening modalities differed not only in diagnostic performance but also in infrastructure requirements and scalability. Simplified age-based or questionnaire-driven approaches required minimal laboratory capacity and were highly scalable, whereas point-of-care testing depended on device availability and supply chains. CHW-led models offered workforce scalability, whereas mHealth-integrated approaches required stable digital infrastructure. These contextual differences are central to determining feasibility across diverse LMIC settings.

Taken together, the evidence supports a pragmatic, resource-stratified framework for the implementation of cardiovascular screening in LMICs. In the most resource-constrained environments, where laboratory services and digital infrastructure are limited, age-based BP screening delivered by trained CHWs may represent the most feasible and scalable entry point. Across multiple studies, CHW-led models demonstrated strong agreement with clinician assessments and high diagnostic reliability, indicating that workforce design is a central determinant of program success. Even simple additions such as weight or BMI can modestly improve risk stratification without substantially increasing operational burden.

Screening program efficiency is also shaped by national prioritization matrices and financing decisions, which, in many LMICs, are determined at the governmental rather than the institutional level.^25^ Alignment between epidemiological evidence and national planning frameworks is therefore essential to ensure efficient resource allocation.

As diagnostic capacity expands, combining BP screening with capillary or fasting glucose testing can strengthen detection of cardiometabolic risk while remaining practical. In these contexts, locally calibrated and simplified risk tools may provide a more appropriate balance between accuracy and feasibility than complex laboratory-intensive models. However, many risk scores and screening tools have not undergone external validation or formal recalibration within diverse LMIC populations. Without such validation, predictive performance may deteriorate when applied across different epidemiological and demographic contexts. Future programs should prioritize local validation before scale-up.

Importantly, screening effectiveness appears to improve when cardiovascular risk assessment is embedded within existing care platforms rather than delivered as standalone campaigns.^26^ Integration into routine primary care visits, HIV clinics, or established chronic disease programs may enhance uptake, reduce missed opportunities, and facilitate completion of referrals by leveraging established patient-provider relationships. CHWs can play a central role in this integration by supporting patient education, follow-up, and navigation across levels of care.

Where mobile or digital infrastructure is available, simplified risk models can be incorporated into community-based workflows to strengthen documentation, supervision, and referral tracking. However, expansion to more sophisticated models should be guided not only by diagnostic capability but by broader system readiness, including medication availability, laboratory capacity, continuity of care, and sustained workforce support.

Globally, these findings support a shift away from isolated vertical screening campaigns toward integrated, system-oriented cardiovascular prevention strategies tailored to local resource capacity. International agencies, ministries of health, and implementation partners should prioritize scalable models that combine simplified detection, locally validated tools, robust referral systems, and affordable treatment pathways.^27^ Without this broader systems approach, screening alone is unlikely to substantially reduce the growing cardiovascular burden across LMICs.

### Study Limitations

This review has several limitations. First, although the search strategy was broad and included multiple bibliographic databases, gray literature sources, trial registries, and manual reference-list screening, the review was limited to English-language publications. Relevant studies published in other languages may therefore have been missed, particularly given the focus on LMIC settings. Second, the included studies were highly heterogeneous in study design, screening modality, target condition, health care setting, population characteristics, outcome definitions, and follow-up duration. This heterogeneity precluded quantitative meta-analysis and necessitated narrative synthesis. As a result, the findings should be interpreted as a synthesis of patterns across settings rather than pooled estimates of intervention effect. Third, most included studies were observational or program evaluations rather than randomized trials. Several studies were therefore at moderate or serious risk of bias, particularly due to confounding, participant selection, and missing data. These limitations restrict causal inference regarding the effectiveness of specific screening strategies. Fourth, many studies focused on detection yield or diagnostic performance. At the same time, fewer reported downstream implementation outcomes such as confirmatory testing, referral completion, treatment initiation, long-term risk-factor control, cardiovascular events, mortality, or cost-effectiveness. This limits the ability to determine whether screening translated into sustained clinical benefit. Fifth, although no restrictions were placed on participant demographics such as age, sex, or baseline cardiovascular risk, studies were excluded where the population or setting was outside the scope of this review. These included studies conducted exclusively in HICs, studies focused on noncardiovascular screening programs, and hospital-based diagnostic cohorts without a defined population, community, or primary-care screening component. Finally, the review protocol was registered on PROSPERO (International Prospective Register of Systematic Reviews) on 28 November 2025, after the database search had been initiated and completed on 22 November 2025. However, registration occurred before final study selection, data extraction, risk-of-bias assessment, and evidence synthesis were completed. In addition, there was a minor deviation from the registered eligibility criteria. The PROSPERO protocol specified inclusion of studies involving adults aged 18 years and above, whereas the review did not apply a strict age restriction during screening. This decision was made because several LMIC screening programs were implemented at community or population level and reported mixed or broad age groups, making strict exclusion by age difficult and potentially leading to the loss of relevant implementation evidence. However, this deviation may have increased clinical heterogeneity across included studies and should be considered when interpreting the findings.

## Conclusions

Cardiovascular screening programs in LMICs can successfully identify substantial unmet cardiometabolic burden, particularly when locally adapted and delivered through community-based models. However, the principal limitation lies in attrition across the care cascade, especially between abnormal screening and confirmatory treatment initiation. Effective screening in LMICs, therefore, depends less on increasingly complex risk algorithms and more on integration with referral systems, simplified tools, and health system strengthening to ensure that detection translates into durable cardiovascular risk reduction.

Perspectives **COMPETENCY IN MEDICAL KNOWLEDGE:** Cardiovascular disease screening programs in low- and middle-income countries can identify substantial previously undiagnosed cardiometabolic risk. Locally validated risk-prediction tools, point-of-care testing, and community health worker–led screening may provide feasible and accurate approaches in resource-constrained settings.

**COMPETENCY IN SYSTEMS-BASED PRACTICE:** Screening effectiveness depends on more than case detection. Programs should incorporate accessible confirmatory testing, structured referral pathways, affordable treatment, and sustained follow-up because the greatest losses frequently occur after an abnormal screening result.

**TRANSLATIONAL OUTLOOK:** Future pragmatic studies should determine which integrated screening-to-treatment models improve referral completion, treatment initiation, long-term risk-factor control, cardiovascular outcomes, and cost-effectiveness. Standardized reporting of each stage of the screening-to-care cascade will facilitate comparison across settings and guide scalable implementation.

## Funding support and author disclosures

Dr Zakkar is supported by the British Heart Foundation award (CH/12/1/29419) to the University of Leicester and Leicester NIHR Biomedical Research Centre (NIHR203327). All other authors have reported that they have no relationships relevant to the contents of this paper to disclose.

## Supplementary materials

Supplementary materials The supplementary materials accompanying this manuscript include Supplementary File 1, which details the comprehensive search strategy used, and Supplementary File 2, which contains the PRISMA 2020 Checklist documenting adherence to the Preferred Reporting Items for Systematic Reviews and Meta-Analyses guidelines.
