# Supplementary material for: Cardiovascular Disease Screening Programs in Low- and Middle-Income Countries: A Systematic Review
Source: JACC Adv. 2026 Jul 22;5(8):103048. doi: 10.1016/j.jacadv.2026.103048 (PMC13425897; doi:10.1016/j.jacadv.2026.103048)
Supplement: Supplementary materials — The supplementary materials accompanying this manuscript include Supplementary File 1, which details the comprehensive search strategy used, and Supplementary File 2, which contains the PRISMA 2020 Checklist documenting adherence to the Preferred Reporting Items for Systematic Reviews and Meta-Analyses guidelines. [file mmc1.pdf]

Supplementary File S1. Literature Search Strategy

Databases searched

The following electronic databases were systematically searched from database inception to 22 November 2025:

- MEDLINE (via PubMed)
- EMBASE
- CINAHL
- Web of Science Core Collection
- Cochrane Central Register of Controlled Trials (CENTRAL)

MEDLINE (PubMed) Search Strategy

("Cardiovascular Diseases"[Mesh] OR "cardiovascular disease*"[tiab] OR "heart disease*"[tiab] OR cardiometabolic[tiab]
OR hypertension[tiab] OR "high blood pressure"[tiab] OR diabetes[tiab] OR "cardiovascular risk"[tiab])
AND
("Mass Screening"[Mesh] OR screening[tiab] OR "risk assessment"[tiab] OR "risk prediction"[tiab]
OR "risk score"[tiab] OR "population screening"[tiab] OR "community screening"[tiab] OR "early detection"[tiab])
AND
("Developing Countries"[Mesh] OR "low income countr*"[tiab] OR "middle income countr*"[tiab] OR LMIC*[tiab]
OR "resource limited"[tiab] OR "resource constrained"[tiab] OR "low resource setting*"[tiab]
OR Africa[tiab] OR Asia[tiab] OR "Latin America"[tiab])

EMBASE Search Strategy

('cardiovascular disease'/exp OR 'cardiovascular disease*':ti,ab OR 'heart disease*':ti,ab
OR hypertension:ti,ab OR diabetes:ti,ab OR 'cardiometabolic risk':ti,ab)
AND
('mass screening'/exp OR screening:ti,ab OR 'risk assessment':ti,ab OR 'risk prediction':ti,ab

OR 'risk score':ti,ab OR 'population screening':ti,ab OR 'community screening':ti,ab)
AND
('developing country'/exp OR 'low income countr*':ti,ab OR 'middle income countr*':ti,ab
OR LMIC*:ti,ab OR 'resource limited':ti,ab OR 'low resource setting*':ti,ab)

CINAHL Search Strategy

(MH "Cardiovascular Diseases" OR TI cardiovascular disease* OR AB cardiovascular disease*
OR TI hypertension OR AB hypertension OR TI diabetes OR AB diabetes)
AND
(MH "Mass Screening" OR TI screening OR AB screening OR TI risk assessment OR AB risk assessment)
AND
(MH "Developing Countries" OR TI LMIC* OR AB LMIC* OR TI "low income countr*"
OR AB "low income countr*" OR TI "middle income countr*" OR AB "middle income countr*")

Web of Science Search Strategy

TS = (
("cardiovascular disease*" OR "heart disease*" OR hypertension OR diabetes OR "cardiometabolic risk")
AND
(screening OR "risk assessment" OR "risk prediction" OR "population screening" OR "community screening")
AND
("low income countr*" OR "middle income countr*" OR LMIC* OR "developing countr*" OR "resource limited")
)

Cochrane CENTRAL Search Strategy

("cardiovascular disease*" OR hypertension OR diabetes OR "cardiometabolic risk")
AND

(screening OR "risk assessment" OR "risk prediction" OR "population screening")
AND
("low income countr*" OR "middle income countr*" OR LMIC* OR "developing countr*")

PRISMA

PRISMA 2020 Checklist

Section and Topic	Item #	Checklist item	Location where item is reported
**TITLE**			
Title	1	Identify the report as a systematic review.	Title
**ABSTRACT**			
Abstract	2	See the PRISMA 2020 for Abstracts checklist.	
**INTRODUCTION**			
Rationale	3	Describe the rationale for the review in the context of existing knowledge.	Introduction
Objectives	4	Provide an explicit statement of the objective(s) or question(s) the review addresses.	Introduction
**METHODS**			
Eligibility criteria	5	Specify the inclusion and exclusion criteria for the review and how studies were grouped for the syntheses.	Eligibility Criteria
Information sources	6	Specify all databases, registers, websites, organisations, reference lists and other sources searched or consulted to identify studies. Specify the date when each source was last searched or consulted.	Search Strategy
Search strategy	7	Present the full search strategies for all databases, registers and websites, including any filters and limits used.	Supplementary materials
Selection process	8	Specify the methods used to decide whether a study met the inclusion criteria of the review, including how many reviewers screened each record and each report retrieved, whether they worked independently, and if applicable, details of automation tools used in the process.	Search Strategy
Data collection process	9	Specify the methods used to collect data from reports, including how many reviewers collected data from each report, whether they worked independently, any processes for obtaining or confirming data from study investigators, and if applicable, details of automation tools used in the process.	Data Extraction
Data items	10a	List and define all outcomes for which data were sought. Specify whether all results that were compatible with each outcome domain in each study were sought (e.g. for all measures, time points, analyses), and if not, the methods used to decide which results to collect.	Data Extraction
	10b	List and define all other variables for which data were sought (e.g. participant and intervention characteristics, funding sources). Describe any assumptions made about any missing or unclear information.	Data Extraction
Study risk of bias assessment	11	Specify the methods used to assess risk of bias in the included studies, including details of the tool(s) used, how many reviewers assessed each study and whether they worked independently, and if applicable, details of automation tools used in the process.	Study selection
Effect measures	12	Specify for each outcome the effect measure(s) (e.g. risk ratio, mean difference) used in the synthesis or presentation of results.	Data Synthesis
Synthesis methods	13a	Describe the processes used to decide which studies were eligible for each synthesis (e.g. tabulating the study intervention characteristics and comparing against the planned groups for each synthesis (item #5)).	Risk of Bias and Quality Assessment
	13b	Describe any methods required to prepare the data for presentation or synthesis, such as handling of missing summary statistics, or data conversions.	Data Synthesis
	13c	Describe any methods used to tabulate or visually display results of individual studies and syntheses.	Data Synthesis
	13d	Describe any methods used to synthesize results and provide a rationale for the choice(s). If meta-analysis was performed, describe the model(s), method(s) to identify the presence and extent of statistical heterogeneity, and software package(s) used.	Data Synthesis
	13e	Describe any methods used to explore possible causes of heterogeneity among study results (e.g. subgroup analysis, meta-regression).	Not applicable
	13f	Describe any sensitivity analyses conducted to assess robustness of the synthesized results.	Not applicable
Reporting bias	14	Describe any methods used to assess risk of bias due to missing results in a synthesis (arising from reporting biases).	Study selection

PRISMA 2020 Checklist

Section and Topic	Item #	Checklist item	Location where item is reported
assessment			
Certainty assessment	15	Describe any methods used to assess certainty (or confidence) in the body of evidence for an outcome.	Risk of Bias and Quality Assessment
**RESULTS**			
Study selection	16a	Describe the results of the search and selection process, from the number of records identified in the search to the number of studies included in the review, ideally using a flow diagram.	Figure 2
	16b	Cite studies that might appear to meet the inclusion criteria, but which were excluded, and explain why they were excluded.	Not applicable
Study characteristics	17	Cite each included study and present its characteristics.	Table 2
Risk of bias in studies	18	Present assessments of risk of bias for each included study.	Table 1/ Figure 1
Results of individual studies	19	For all outcomes, present, for each study: (a) summary statistics for each group (where appropriate) and (b) an effect estimate and its precision (e.g. confidence/credible interval), ideally using structured tables or plots.	Not applicable
Results of syntheses	20a	For each synthesis, briefly summarise the characteristics and risk of bias among contributing studies.	Not applicable
	20b	Present results of all statistical syntheses conducted. If meta-analysis was done, present for each the summary estimate and its precision (e.g. confidence/credible interval) and measures of statistical heterogeneity. If comparing groups, describe the direction of the effect.	Not applicable
	20c	Present results of all investigations of possible causes of heterogeneity among study results.	Risk of Bias and Quality Assessment
	20d	Present results of all sensitivity analyses conducted to assess the robustness of the synthesized results.	Not applicable
Reporting biases	21	Present assessments of risk of bias due to missing results (arising from reporting biases) for each synthesis assessed.	Not applicable
Certainty of evidence	22	Present assessments of certainty (or confidence) in the body of evidence for each outcome assessed.	Not applicable
**DISCUSSION**			
Discussion	23a	Provide a general interpretation of the results in the context of other evidence.	Discussion
	23b	Discuss any limitations of the evidence included in the review.	Limitations
	23c	Discuss any limitations of the review processes used.	Limitations
	23d	Discuss implications of the results for practice, policy, and future research.	Conclusion
**OTHER INFORMATION**			
Registration and protocol	24a	Provide registration information for the review, including register name and registration number, or state that the review was not registered.	Methods
	24b	Indicate where the review protocol can be accessed, or state that a protocol was not prepared.	Methods
	24c	Describe and explain any amendments to information provided at registration or in the protocol.	Not applicable
Support	25	Describe sources of financial or non-financial support for the review, and the role of the funders or sponsors in the review.	Funding
Competing interests	26	Declare any competing interests of review authors.	Not applicable
Availability of	27	Report which of the following are publicly available and where they can be found: template data collection forms; data extracted from	Data availability

Section and Topic	Item #	Checklist item	Location where item is reported
data, code and other materials		included studies; data used for all analyses; analytic code; any other materials used in the review.	statement

From: Page MJ, McKenzie JE, Bossuyt PM, Boutron I, Hoffmann TC, Mulrow CD, et al. The PRISMA 2020 statement: an updated guideline for reporting systematic reviews. BMJ 2021;372:n71. doi: 10.1136/bmj.n71.
This work is licensed under CC BY 4.0. To view a copy of this license, visit https://creativecommons.org/licenses/by/4.0/

Study (Author, Year)	Country/Setting	Primary reason for exclusion	Clarification
Maurer & Ramos 2015[27]	Multi-country LMIC analysis (WHO SAGE dataset)	Ineligible study design/intervention scope	Secondary analysis modelling the potential impact of opportunistic hypertension screening using survey data rather than evaluation of an implemented cardiovascular screening programme with measurable screening-performance or implementation outcomes
Dukpa et al. 2015[28]	Bhutan primary care setting	Ineligible study design	Model-based economic evaluation assessing cost-effectiveness of diabetes and hypertension screening strategies rather than a primary empirical study evaluating implementation or real-world effectiveness of a cardiovascular screening programme
Akinyemi et al. 2023[29]	Nigeria antenatal tertiary hospital setting	Ineligible population/screening focus	Focused exclusively on gestational diabetes screening in pregnant women rather than broader cardiovascular disease or cardiometabolic screening programmes in general adult LMIC populations aligned with the review objectives
